# The Impact of Oil Type on the Performance of β-Amyrin-Based Oleogels: Formation, Physicochemical Properties, and Potential Correlation Analysis

**DOI:** 10.3390/foods13060876

**Published:** 2024-03-14

**Authors:** Shuxian Su, Si Qin, Huiping Xia, Peiwang Li, Haiyan Li, Chenjia Li, Shiyin Guo, Chaoxi Zeng

**Affiliations:** 1 Department of Food Science and Technology, College of Food Science and Technology, Hunan Agricultural University, No. 1 Nongda Road, Furong District, Changsha 410128, China; sushuxian1118@stu.hunau.edu.cn (S.S.); qinsiman@hunau.edu.cn (S.Q.); hpxia@hunau.edu.cn (H.X.); 13578374900@stu.hunau.edu.cn (H.L.); chenjiali@stu.hunau.edu.cn (C.L.); 2 State Key Laboratory of Utilization of Woody Oil Resource, Hunan Academy of Forestry, 658 Shaoshan South Road, Tianxin District, Changsha 410128, China; 17184655@163.com; 3 College of Life Sciences and Health, Hunan University of Science and Technology, No. 1 Taoyuan Road, Xiangtan 411201, China

**Keywords:** β-amyrin, oleogel, fatty acid properties, stability of the oleogels, correlational analysis

## Abstract

Pentacyclic triterpenes show potential as oleogelators, but their combination with various vegetable oils has limited research. This study selected linseed, rapeseed, sunflower, coconut, and palm oils to combine with the triterpenoid compound β-amyrin for the preparation of oleogels. The stability, crystal network structure, and other properties of each oleogel were evaluated. The correlation between different oil types and the properties of corresponding oleogels was explored. The results showed that β-amyrin formed stable oleogels with five vegetable oils under suitable temperature conditions, wherein especially the LO-based oleogel not only exhibited higher oil-binding capacity and hardness, but also demonstrated excellent stability at the microscopic level and notable rheological properties. Further analysis revealed a close correlation between the physicochemical properties of the oleogels and lipid characteristics, indicating that oleogels prepared from long-chain highly unsaturated fatty acids exhibit high stability. The above results indicate that β-amyrin can be a novel candidate oleogelator and that the oil type can modify the properties of β-amyrin-based oleogels. This study provides the latest reference for the application of pentacyclic triterpenoids in food.

## 1. Introduction

Foods made of oils and fats provide an important energy source for the human body. A network of fat crystals composed of trans fats and saturated fats produced by hydrogenation, transesterification, or blending comprises a large market share of margarine, chocolate, and other foods rich in solid fats [1,2]. Due to excessive intake of saturated fatty acids and trans fatty acids, cardiovascular disease is induced by increased levels of low-density lipoprotein (LDL), and the risk of type II diabetes mellitus is increased by inducing insulin resistance [3,4,5]. To be more in line with the contemporary concept of healthy eating and to promote the progress of the energy-saving food market, new technologies or methods should be used to prepare products that are comparable to traditional solid fats.

Oleogels preparation, a technique that imparts solid characteristics to liquid vegetable oils, has emerged as a novel and efficient approach to prepare solid fats. It can enhance not only the product’s qualities such as shelf life, but also improve the quality of food nutrition and nutritional value [4,6,7]. The oleogelator used to make oleogels can be classified into two categories: small molecule structuring agents and large molecule structuring agents, depending on the relative molecular mass of the oleogelator. The first category comprises natural waxes, glycerol esters, fatty acids, fatty alcohols, lecithin, mono- and diglycerides of glycerol, and similar substances [1,8,9]. The second category includes polymers and proteins and their derivative structural agents such as proteins, ethyl cellulose, hydroxypropyl methyl cellulose, methyl cellulose, chitosan, chitin, and so on [10,11,12]. Presently, numerous researchers have investigated diverse applications of oleogels in food production, such as for alternatives to margarine [13], as well as the creation of functional chocolates [14], ice creams [15], cakes [16], and more. Nevertheless, the existing research on oleogels falls short of fully addressing the present requirements for practical use, mostly due to the limited variety of oleogelators that are currently available. Consequently, the current focus in oleogel research is the active investigation of novel oleogelators.

Prior research has indicated that pentacyclic triterpenoids, specifically ursolic acid and oleanolic acid, possess the capability to induce gelation in organic solvents, resulting in the formation of organogels [17,18]. To the best of our knowledge, pentacyclic triterpenoids have not been used to prepare food-grade oleogels. In contrast, β-amyrin, as a class of pentacyclic triterpenoids, is an effective functional ingredient in many food products and traditional Chinese herbal medicines, as well as a characteristic triterpenoid in camellia oil [19]. β-amyrin has anticancer, anti-inflammatory, anti-tumor, anti-diabetic, anti-dyslipidemia, and antidepressant effects [20,21]. It has a wide range of potential uses for healthy food and drug development. Therefore, in this manuscript, we chose to use β-amyrin as an oleogelator for the preparation of oleogels from different vegetable oils to investigate its effect on the properties of the formed oleogels.

The formation of an oleogel is a three-dimensional network structure formed by the interaction of oil with liquid oil during the cooling process, such as hydrogen bonding, van der Waals forces, and dipole–dipole forces. The characteristics of the network structure formed are related to the chain length and unsaturation of the liquid oil [1,22]. Therefore, a large number of studies have examined the effect of different oils on the properties of oleogels, and the results indicate that different oil types are one of the main factors affecting the physicochemical properties of oleogels [23,24,25]. Higher unsaturation increases the crystallinity and network stability of oil-based oleogels, according to Han et al. [1]. Pang et al. found that the degree of unsaturation of the oil phase of the prepared oleogel was proportional to the hardness of the oleogel [13]. Liu et al. discovered that variability in oil polarity due to oil types also affects oleogel performance [26]. Valoppi et al. and Chai et al. pointed out that different types of oils may also affect the formation of the network and thus the final structure of the oleogel due to differences in viscosity [27]. Many studies have analyzed oleogels made from different cooking oils and examined how oil type affects them. However, the effects of different oil types on the properties and mechanisms of the oleogel formed based on triterpenoids such as β-amyrin are still unknown.

This study aimed to investigate the effect of different oil types on the oil properties of β-amyrin gels at different preparation temperatures and to reveal the correlation relationship between different types of oils and the physical properties of the oleogel. In this study, we first examined the possibility of preparing oleogels from β-amyrin and then investigated the probable relationships and correlations that exist between oil types and the physicochemical structure of the oleogels as well as the potential molecular roles during the gelling process of the organogels. This work helps to provide insight into the preparation and properties of pentacyclic triterpenoid oleogels.

## 2. Materials and Methods

### 2.1. Materials

Linseed oil, rapeseed oil, coconut oil, and sunflower oil were purchased from the local market, palm oil was purchased from Yihai Kerry Group, and β-amyrin (purity > 98%) was purchased from Shanxi Jinkangtai Biotechnology Co., Ltd. (Xianyang, China), which can be used directly without purification. All other chemical reagents were analytically pure.

Fatty acid composition was analyzed using gas chromatography. Gas chromatography analysis conditions: using Agilent 7890A gas chromatograph; Agilent J&WDB-FATWAX ultra-high inert column; detector FID; inlet temperature 270 °C, detector temperature 280 °C. Chromatographic column heating program: initial temperature 100 °C, keep 13 min, 10 °C/min to 180 °C, keep 6 min, then 1 °C/min to 200 °C, keep 20 min, then 4 °C/min to 230 °C, keep 10.5 min, until the analysis is complete. The carrier gas is pure nitrogen at a flow rate of 30 cm/s, constant flow mode, hydrogen flow rate of 40 mL/min; air flow rate: 400 mL/min, the tail gas is nitrogen at a flow rate of 25 mL/min, the injection volume is 1 μL, and the sample is injected by shunt mode, the shunt ratio is 100:1. The qualitative analysis of fatty acids in the five samples was conducted by comparing the retention times of fatty acids in the standard reference solution. The quantitative analysis of fatty acid content was performed using the area normalization method in the Agilent Chemstation software, which provided the relative percentage of each fatty acid. The analysis was performed in triplicate for each sample.

### 2.2. Oleogel Preparation

Eight grams of linseed oil, rapeseed oil, palm oil, coconut oil, and sunflower oil were weighed and added with 4% β-amyrin, respectively. The mixture was heated and stirred for 30 min at certain temperatures (60 °C, 80 °C, 100 °C, 120 °C, or 140 °C) with a speed of 500 rpm. All samples were stored at room temperature (25 ± 2 °C) for 24 h before detection. The gelability of the samples was determined by the inverted tube method. Oleogel samples with β-amyrin concentrations of 1–5% (*w*/*w*) were also prepared to determine the critical gel concentrations for different oil phases.

### 2.3. Oil Binding Capacity

The Oil-Binding Capacity (OBC) of the oleogel was analyzed according to the method described by Da et al. [28]. The oleogel samples prepared under various temperature conditions were collected in 1.5 mL centrifuge tubes. Following high-speed centrifugation at 13,000 rpm for 10 min, the tubes were inverted onto filter paper and allowed to stand for 30 min to ensure complete drainage of any remaining liquid oil. The OBC was calculated using the provided formula 1, and the experiment was repeated three times with the average of the results taken. Additionally, the oleogel samples were kept at room temperature for 0, 14, and 28 days to compare the differences in OBC among the different samples.

(1) $$OBC=-\frac{c-a}{b-a}\;\times \mathrm{100}\%$$

where *c* denotes the weight of the oleogel and tube after centrifugation (g), *b* denotes the weight of the oleogel and tube before centrifugation (g), and *a* denotes the weight of the empty tube (g).

### 2.4. Texture Analysis

A texture analyzer (TA. XT Plus, Surrey, UK) test was performed to determine the textural properties of the oleogels [29]. The samples prepared at 80 °C, 100 °C, and 120 °C were left at 25 °C for 24 h before measurement. The texture analyzer was set with the parameters of a test speed of 1 mm/s and a compression distance of 10.00 mm. Each sample was tested three times, and the average value was taken. The oleogel samples were then kept at 25 °C for 0, 14, and 28 days, followed by hardness measurements under the same conditions.

### 2.5. Polarized Light Microscopy

The microstructure of the oleogel was observed using a polarized microscope (Olympus, BX53M, Tokyo, Japan). To observe the crystalline morphology of the oleogel at different heating temperatures, the samples were photographed at 25 °C using a 100× magnification (10× eyepiece and 10× objective) under the polarizing microscope. An appropriate amount of the oleogel was taken and placed on a clean slide. The slide was then covered to ensure even distribution of the oleogel.

ImageJ software (NIH, Bethesda, MD, USA) with the FracLac plugin was used to calculate fractal dimension (Db), average length (L), and lacunarity (A) in two-dimensional space for quantitative analysis. The box-counting method was employed to calculate these parameters. The formula for the box-counting method is as follows:

(2) $$Db=-\frac{logN\varepsilon }{log\varepsilon }$$

### 2.6. X-ray Diffraction Analysis

The crystalline shape of the oleogel samples was analyzed by the X-diffraction technique (XRD-6000 type, Shimadzu, Kyoto, Japan) by referring to the method of Blake et al. [30]. The instrument test conditions were set as follows: Cu target as the radiation source (wavelength λ = 1.5401 Å), operating voltage 40 kV, operating current 40 mA, scanning step size 0.02°, scanning rate 2°/min, emitting and antireflecting slit 1.0 mm, receiving slit 0.1 mm, test temperature 25 °C, and scanning range of 5.0–55.0° at 2θ angle.

### 2.7. Differential Scanning Calorimetry

Samples were prepared for differential scanning calorimetry (DSC-822, Mettler Toledo, Zurich, Switzerland) measurements by placing 5 mg of oleogel in an aluminum pan. Each sample was kept at 120 °C for 5 min until completely melted to eliminate crystal memory. The temperature was reduced to −65 °C of cooling rates of 5 °C/min and 10 °C/min. After holding for 5 min, the temperature was raised to 30 °C at the same heating rate. Melting and crystallization curves of the samples under different oil treatments were recorded. The onset dissolution temperature (Ton-m), the melting peak temperature (Tm), the onset crystallization temperature (Ton-c), and the crystallization peak temperature (Tc) of the samples were measured utilizing the DSC curves provided by the TA Instruments Universal Analysis Software. All measurements were repeated three times.

### 2.8. The Crystallization Kinetics of Oleogel

The heat flow rate obtained during the crystallization process during DSC measurements was integrated against time using Origin software. The Avrami equation was used to study the crystallization kinetics of the oleogel under isothermal conditions, and the relative crystallinity X(t) at time t was obtained, which is of the following form:

(3) $$X\left(t\right)=1-exp\left(-kt^{n}\right)$$

where X(t) is the crystallinity at time t; t is the crystallization time; k is the complex crystallization rate constant; and n is the Avrami index.

Equation (1) was obtained by deforming the sample according to its relative crystallinity X(t):

(4) $$\ln\left[\ln\left(1-Xt\right)\right]=lnk+nlnk$$

### 2.9. Rheological Measurement

Analysis of the rheological behavior of the oleogels was performed using a rotational rheometer (Kinexus Pro+ Rheometer, Malvern, London, UK). Strain scanning experiments were carried out on 40.0 nm diameter flat plates with a plate spacing of 1 mm, a constant frequency of 1 Hz, and a strain range of 0.01–10% at room temperature to determine the linear viscoelastic region. Shear viscosity experiments were carried out at shear rates between 0.1–100 s^–1^. Frequency scanning experiments were carried out at frequencies between 0.01 and 10 Hz with a fixed strain and the rheological behavior of the oleogel was analyzed using the power law equation.

G′ = a (ω)^b^ (5)

where G′ is the storage modulus (Pa), a (Pa·b) is a parameter used to describe the rheological behavior, ω is the frequency (rad/s), and b is a dimensionless flow behavior index.

### 2.10. Statistical Analysis

Significant differences between means were determined using Duncan’s analysis of variance (ANOVA), and multiple comparisons between treatments were analyzed using ANOVA with significant differences at the 5% level (*p* < 0.05). Each experiment was repeated thrice, and results were expressed as mean ± standard deviation. Statistical analysis was performed using Origin 2021.

## 3. Results and Discussion

### 3.1. Concentration Phase Diagrams

**Figure 1:**
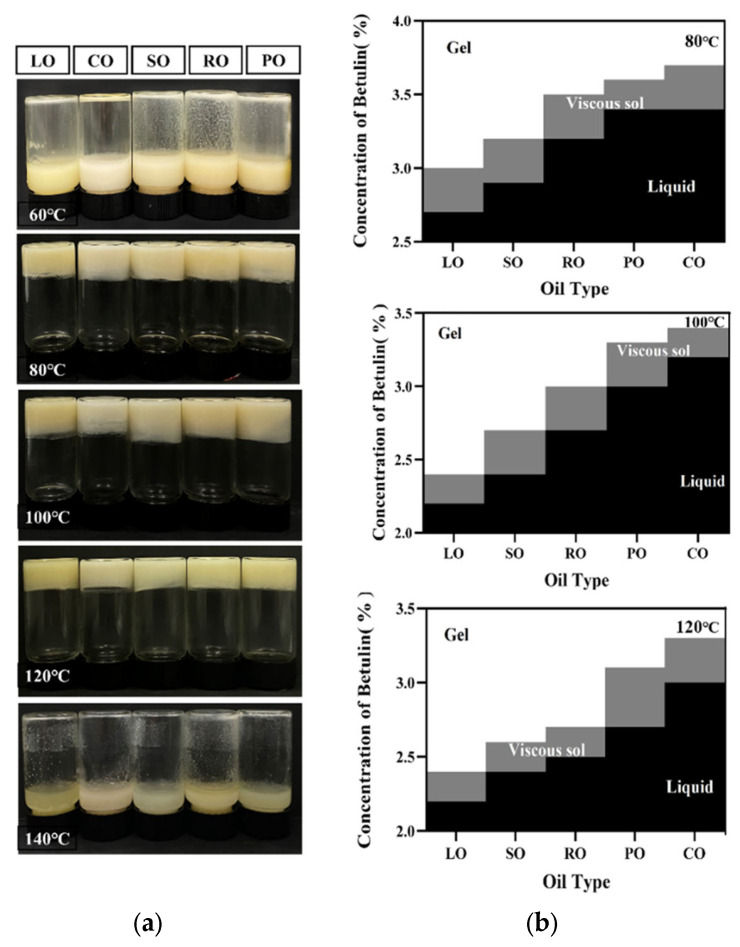
Gel property analysis of β-amyrin-based oleogel. (**a**) The appearance of 4% β-amyrin-based oleogels at different preparation temperatures; (**b**) shows the qualitative phase diagram of different concentrations of β-amyrin-based oleogel under different oil conditions: liquid (no gelation occurred), viscous sol (viscous solution, with fluidity), and gel (i.e., free-standing gel).

This study investigated how different types of oils influenced the oil phase transition content in β-amyrin oleogel at various preparation temperatures. The results showed that β-amyrin was successfully used to prepare oleogels at high and appropriate temperatures. Additionally, various oils exhibited similar oleogel formation trends at different temperatures (Figure 1a). When the preparation temperature is below 80 °C, the samples did not exhibit any thickening or gelation, and the formation of a gel-to-oil phase was not feasible. This lack of oleogel formation can be attributed to the fact that β-amyrin is unable to establish a stable three-dimensional network structure at lower temperatures, thereby struggling to impede the flow of liquid oil [31,32]. As the preparation temperature rises, the intermolecular forces lead to the aggregation of β-amyrin particles, forming a three-dimensional network structure. This network structure hinders the flow of lipophilic liquid, resulting in the gelation of the entire system [33]. However, when the temperature reaches 140 °C, the increase in temperature causes the unsaturated fatty acids in the vegetable oil to be oxidized and degraded [34].

The minimum concentration required to form an oleogel reflects the gelling ability of β-amyrin in different types of oils. The temperature during the preparation process affects the critical concentration required to form an oleogel. At a preparation temperature of 80 °C (Figure 1b), the critical concentration of β-amyrin for LO-based oleogel is 3%, while for SO and RO, it is 3.2% and 3.5%, respectively. The highest critical concentration is observed for CO at 3.7%. With an increase in the preparation temperature, there is an overall decreasing trend in the critical concentration of gel-forming oil. Consequently, within a specific temperature range, higher temperatures necessitate lower critical concentrations for the formation of an oleogel, implying a reduced quantity of gelating agent necessary to achieve gelation.

The type of oil significantly influences the critical concentration required for the formation of oleogels. At a preparation temperature of 100 °C, compared to other oil types, the critical concentration for oleogels formation in RO and CO are reduced to 3.0% and 3.4%, respectively. LO has the lowest β-amyrin concentration, with the essential concentration for a stable oleogel being 2.4%. From 100 °C to 120 °C, the viscous sol-gel state distribution range rose substantially. Other than for LO, which showed no change, the other four oils showed a slightly decreasing trend in critical concentration, but LO still had the lowest gelling concentration and PO and CO had greater critical concentrations. This result is consistent with the findings of Han et al. [1] and may be attributed to the higher content of linoleic acid in LO, which has the highest degree of unsaturation. This high unsaturation level is favorable for β-amyrin to undergo one-dimensional growth, helix formation, and twisting, resulting in a more stable and robust three-dimensional network structure [33,35,36,37]. On the other hand, CO and PO are rich in short-chain saturated fatty acids, which overall decrease the ability to bind oils and lower the critical concentration for gel formation. Therefore, the gel network structure formed by the self-assembly of oils rich in long-chain unsaturated fatty acids and β-amyrin in a binary system exhibits enhanced stability at the appropriate preparation temperature.

### 3.2. Oil Binding Capacity and Hardness Analysis

**Figure 2:**
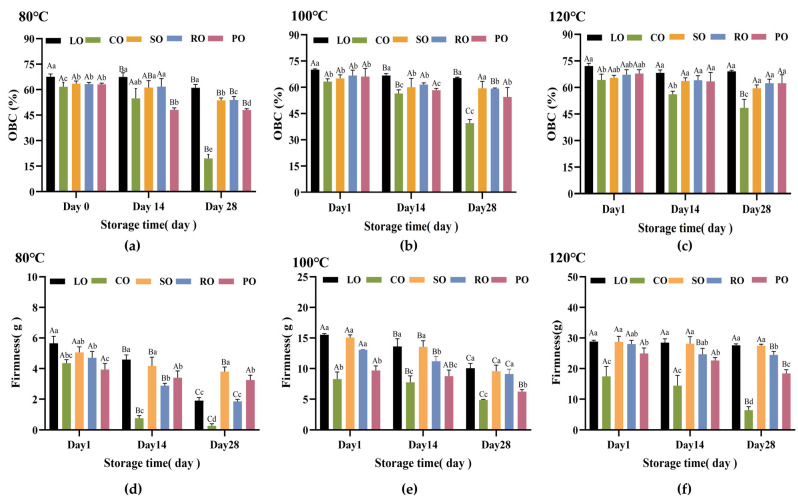
Physical stability of oleogels. (**a**–**c**) The changes in oil binding capacity values of oleogels prepared from 4% β-amyrin with LO, SO, RO, PO, and CO at temperatures of 80 °C, 100 °C, and 120 °C for 0, 14, and 28 days. (**d**–**f**) Hardness values of oleogels prepared from 4% β-amyrin with LO, SO, RO, PO, and CO at 80–120 °C temperatures on days 1, 14, and 28. Different capital letters indicate significant differences between the same samples at different stor-age times (*p* < 0.05). Different lowercase letters indicate significant differences between samples of different oil types for the same storage time (*p* < 0.05).

Hardness and Oil Binding Capacity (OBC) are used to evaluate the ability of oleogels to achieve structural properties similar to solid fat using a minimal amount of oleogelators, without significantly impacting the sensory characteristics of the product [38]. As temperature rises, oleogel OBC increases significantly (Figure 2a–c). The network structure of an oleogel may catch more liquid oil as the temperature rises, increasing OBC. Figure 2d–f indicates a significant increase in hardness as the preparation temperature rises. Particularly, at a preparation temperature of 80 °C, the CO-based oleogel, which is rich in saturated fatty acids, exhibits a low hardness value of 4.35 g. However, at a preparation temperature of 120 °C, the hardness value of the CO-based oleogel increases to 17.44 g. In addition, according to Figure S1, it can be concluded that the correlation coefficient between temperature and hardness values is greater than 0.98, showing a linear positive correlation. The reason for this could be that as the temperature increases, during the same cooling process, non-covalent bonds spontaneously assemble into aggregates, increasing the entanglement of the three-dimensional network formed by β-amyrin. Therefore, the flow of liquid oil is restricted, leading to the appearance of solid-like properties.

The hardness and OBC of oleogels are both significantly influenced by the unsaturation and the fatty acid chain length of the oils [32]. At a preparation temperature of 120 °C, the hardness values of oleogels prepared with LO, SO, and CO are 28.79 g, 28.73 g, and 17.44 g, respectively. The corresponding OBC values are 72.08%, 65.47%, and 59.59%, respectively. According to Table S1, the oleogel formed by C18:3-dominated LO is more tightly structured compared to oleogels prepared from other oil types and thus has significantly higher hardness [39]. This can be attributed to the unique conformation of the C18:3, which enables it to be more tightly encapsulated within the β-amyrin network. Additionally, the presence of polyunsaturated fatty acids promotes chain–chain interactions between the β-amyrin molecules, enhancing the structural and physical stability of the oleogel [4].

On the other hand, the oil type can also affect the physical properties of the oleogel through polarity differences. According to Table S2, it can be concluded that the total polar component (TPC) content is higher in LO at 16% [23], while it is only 4% in CO [40]. That finding provides evidence of a positive correlation between TPC and the degree of oil unsaturation. It may be because oils enriched with higher levels of polar compounds increase the thermally induced oxidation process, promoting the formation of hydrogen bonds between the solvent and the oleogelator. This contributes to the structural integrity of the gel matrix, thereby improving the stability of the textural morphology of the oleogel.

Oil types can affect the storage stability of oleogels. As the storage time increases, the OBC and hardness exhibit a decline at 80 °C and 100 °C. Notably, the OBC of the CO-based oleogel decreases significantly, potentially due to its higher content of saturated fatty acids [41]. At a preparation temperature of 120 °C, the OBC and hardness of the LO-based oleogel remain relatively stable as the storage time increases. This finding aligns with the reported literature, which suggests a positive correlation between the degree of unsaturation in oils and the stability of oleogel hardness [33,42]. In conclusion, the OBC and hardness tended to be consistent over a certain storage time, and the fatty crystal network structure of the oleogel prepared from the long-chain oil type with high unsaturation had the best mechanical strength and better storage stability.

### 3.3. Polymorphism of Different Oleogels

**Figure 3:**
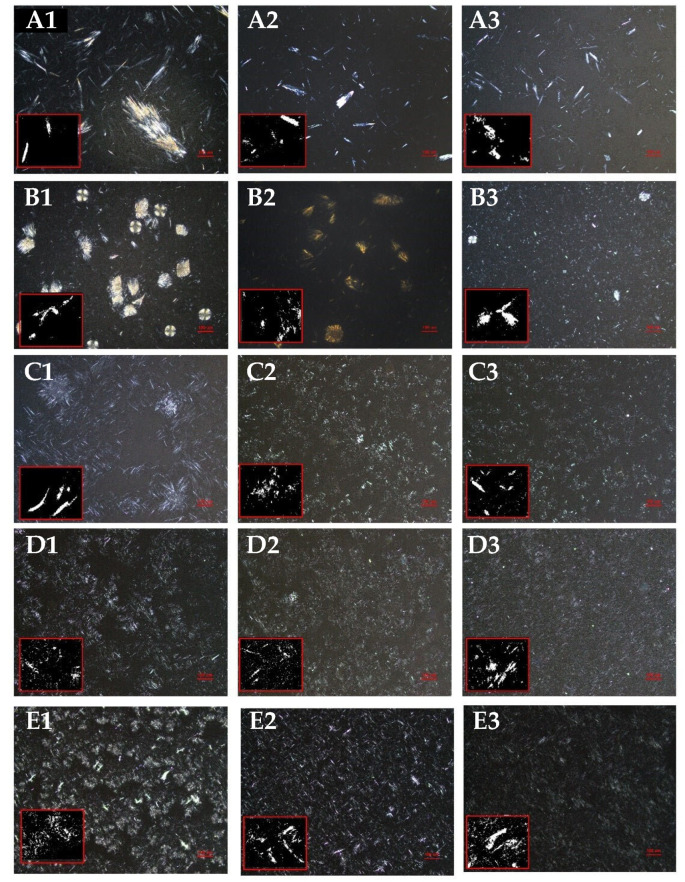
Microphotographs of oleogels prepared at different temperature conditions (80 °C, 100 °C, 120 °C). (**A1**–**A3**) CO-based oleogel, (**B1**–**B3**) PO-based oleogel, (**C1**–**C3**) RO-based oleogel, (**D1**–**D3**) SO-based oleogel, (**E1**–**E3**) LO-based oleogel. Scale bar: 100 µm.

The microscopic images of the oleogel were obtained using polarized light microscopy in Figure 3, aiming to explore the impact of different oil types on the microstructure of the β-amyrin oleogel’s crystal network. As shown in the image, oleogel crystal shape and size differ at 80 °C. The CO-based oleogel has irregular long needle-like fibrous aggregated crystals, while the PO-based oleogel has bigger spherical crystals. The RO-based oleogel has a chrysanthemum shape, and the SO and LO-based oleogels have fine needle-like aggregated structures like those described by Frolova et al. [33]. Interestingly, at a preparation temperature of 100 °C, there is a significant alteration in the crystal morphology of the PO-based oleogel, with a predominant presence of uneven fibrous aggregates. As the preparation temperature is raised to 120 °C, all of the oleogels, except for the CO-based oleogel, exhibit similar features in terms of crystal morphology and arrangement. They undergo a transition from relatively dispersed crystals to a dense and uniformly stable crystal network, with consistent spacing between the crystals. In conclusion, as the preparation temperature increases, the crystal types of different oleogels transform into needle-like and fine fibrous structures, with the crystals gradually distributed more uniformly.

Table 1 analyzes the fractal dimension (Db), average length (L), and lacunarity (A) of microscopic structures in different oleogels, investigating how oil types influence the uniformity of crystal network distribution in the oleogels. Smaller L-values mean better OBC of oleogel internal crystals and more oil-binding surface area. Larger Db values allow for quick nucleation and production of more crystals inside of the oleogel, resulting in smaller, denser, uniformly distributed crystal clusters that confine more liquid oil to a denser, more uniform crystal network. When the Db value is smaller and the A value is larger, crystals tend to aggregate, resulting in wider crystal spacing. This hinders the connectivity of the crystal network and results in weak interparticle interactions between β-amyrin particles [27,43]. Over time, this may decrease the hardness of the oleogel, decrease the OBC, and lead to phase separation of the oleogel [44,45].

Based on the results in Table 1, the internal arrangement of crystals in the oleogels is temperature dependent. As the preparation temperature increases, the Db value increases gradually, while the A and L values decrease. This can be attributed to variations in the van der Waals forces between oils and β-amyrin molecules, resulting in differences in the size and structure of the β-amyrin crystals [1]. When comparing oleogels formed by different oil types at the same preparation temperature, it was observed that the LO-based oleogel had the smallest A and L and the largest Db value. This can be explained by the fact that, compared to oleogels prepared with other oils, the LO-based oleogel contains a higher amount of polyunsaturated fatty acids. This leads to smaller and more uniformly distributed crystal clusters in the oleogel, making it easier to achieve a stable gel effect. This further supports the reason for the changes in storage stability results.

Comparing the XRD spectra of β-amyrin (Figure S2), different oils, and β-amyrin gels based on various oil types (Figure S3), it is evident that the long-spacing peaks of β-amyrin diminish or vanish when it is dissolved in different edible oils. This can be attributed to the rearrangement and recrystallization of β-amyrin crystals as they aggregate in the presence of oils. Comparing the oleogel results of different oil types, it was observed that the amorphous peak areas were similar among the five oleogels in the wide-angle range (2θ > 15°). However, the d-spacing of the oleogels varied, with the LO-based oleogel having the shortest d-spacing and the CO-based oleogel having the highest d-spacing. The observed phenomenon may be attributed to the distorted spatial structure of unsaturated fatty acid molecules in long chains, which affects the electron absorption capacity of the carbonyl group. As a result, a higher number of methyl groups exhibit asymmetric stretching and vibration, leading to the LO-based oleogel having stronger van der Waals forces and forming a stable oleogel [46]. Crystal morphology analysis revealed that different oleogels displayed a prominent diffraction peak at 2θ = 19.93 (corresponding to a d-spacing of 4.42 Å–4.6 Å). This suggests that the triglycerides in the oleogels primarily adopt a β-form monoclinic subcell structure [29]. It is likely that the interaction between the triglyceride molecules and dissolved β-amyrin in the oil induces a modification of the crystal morphology [47]. By comparing the relative crystallinity (RC) of the oleogels at different preparation temperatures (Table S2), it is evident that, except for the CO-based oleogel, which shows insignificant changes in RC, the relative crystallinity of the other oleogels tends to increase. This observation suggests that the difference in crystallinity is primarily due to the degree of exposure of alkyl groups in the liquid oil, which may be related to the spatial structure of the molecules formed through crystal cross-linking and entanglement during the gelation process [41]. Additionally, at a preparation temperature of 120 °C, the relative crystallinity of 4 wt% CO (70.87%) and PO (77.85%) is lower compared to 4 wt% LO (86.91%) and 4 wt% RO (79.21%). This can be attributed to the higher degree of unsaturation in the liquid oils, resulting in a higher degree of crystallinity, which is in line with faster crystal growth rates in crystallization kinetics.

In summary, the various characteristic indices reflected by XRD patterns are influenced by different oil types and preparation temperatures in oleogels. At a certain preparation temperature, the higher the degree of unsaturation of long-chain fatty acids in different vegetable oils, the stronger the effect of the bent spatial arrangement, resulting in greater mechanical strength of the formed oleogels.

### 3.4. Thermal Behaviour of Oleogels

**Figure 4:**
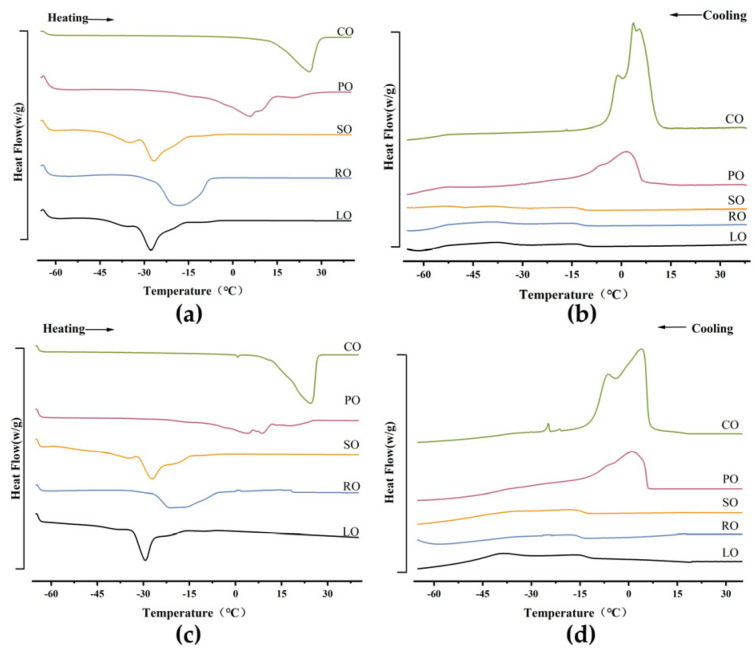
Melting and crystallization curves of different oleogels at 120 °C preparation temperature. (**a**,**b**) Melting and crystallization curves of LO—based oleogels, CO—based oleogels, SO—based oleogels, RO—based oleogels, and PO—based oleogels at 10 °C/min. (**c**,**d**) Melting and crystallization curves of five different oleogels at 5 °C/min.

DSC was used to investigate the thermal behavior of oleogels prepared from different oil types at various cooling rates. The results from Figure 4 and Table S3 indicate that all five oleogels exhibited broad melting and crystallization characteristics. When comparing the Ton values under different crystallization rate conditions, it was observed that both the melting and crystallization peaks became narrower with increasing temperature rates. Additionally, the Tm values under melting conditions shifted towards higher temperatures as the temperature rate increased. This shift is likely due to the rapid melting of crystals at higher temperature rates, consistent with the findings of Han et al. [1]. Moreover, oleogels derived from long-chain unsaturated fatty acids have a broad range of dissolution temperatures. This is attributed to the gradual and continuous transition from a gel to a sol state, rather than an abrupt transformation.

On the other hand, from the crystallization graph, it can be observed that long-chain unsaturated fatty acids exhibit a wide range of crystallization peaks with relatively low peak heights. This is likely due to the β-amyrin molecules behaving as uniformly dispersed amorphous entities within the oil. Interestingly, by comparing the ΔHm (enthalpy of melting) and ΔHc (enthalpy of crystallization) values of oleogels from different oil types, it can be inferred that the thermal stability of oleogels based on LO is poor. This could be attributed to the potential interactions between minor components and unsaturated fatty acids present in the oil formulation, which might affect the melting and crystallization processes.

The crystallization process can be further described using the Avrami model. By fitting the crystallization data to the Avrami model, the values of the Avrami exponent (n) and apparent rate constant (k) were determined, along with the correlation coefficient (R). Based on Table 2, the applied model could adequately describe the experimental data (R^2^ > 0.85), and both the n and k values were influenced by the cooling rate and the type of oil used. The n reflects a parameter related to the time dependence of nucleation and the dimensionality of crystal growth. The n for the oleogels ranged from 2 to 4, indicating a transition from two-dimensional plate-like growth to instantaneous nucleation and spherical crystal growth for the majority of the crystals. Comparing different oleogels, the LO-based oleogel shows the highest n value, indicating an increasing trend with longer chain length. This suggests a transition from spherical to rod-like crystal growth in this oleogel. Additionally, the variation in the value of k indicates changes in the crystal growth rate and nucleation process within the oleogel, resulting in changes in crystal size and type [24,29]. As the cooling rate increases, the k also increases. Specifically, for the LO-based oleogel, k increases from 0.03 to 0.07. This implies that, in a single-component system, samples crystallize faster at higher supercooling temperatures. These results indicate that a higher content of unsaturated fatty acids in the oil phase promotes the formation of more and finer crystals. However, the rate of oleogel formation is not directly correlated with the strength of oleogel formation.

### 3.5. The Rheological Properties of Oloegels

**Figure 5:**
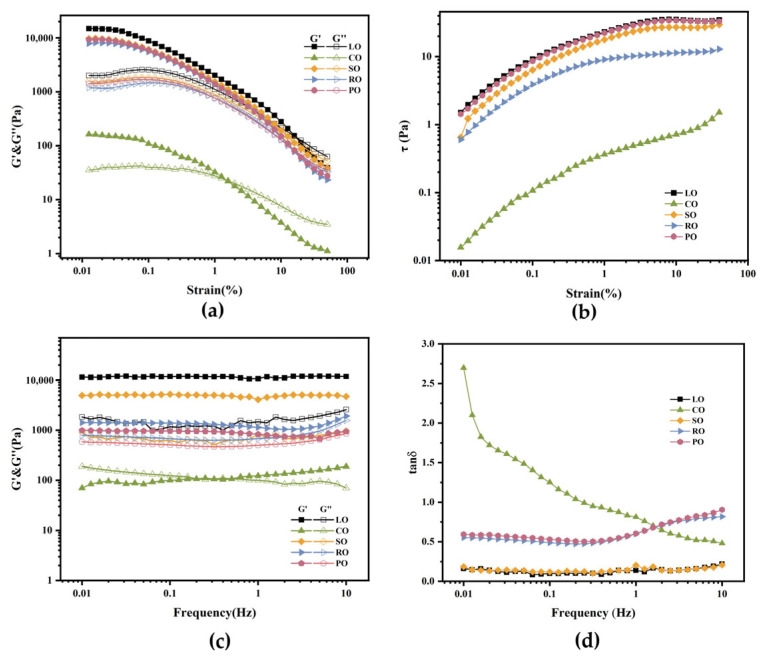
Rheological profiles of oleogels prepared with different oils and 4% β-amyrin under the preparation condition of 120 °C. (**a**) Shear strain sweep curve, (**b**) stress-strain curve, and (**c**,**d**) dynamic frequency sweep plots.

Rheological analysis was conducted on the oleogels to ascertain their relevant properties. As depicted in Figure 5a, within the LVR region, the storage modulus (G′) consistently exceeds the loss modulus (G″), demonstrating that the oleogel prepared with β-amyrin is predominantly a “solid”-dominant sample. Comparing the LO-based oleogel with the CO-based oleogel, the LO-based oleogel exhibits a wider linear viscoelastic region. Additionally, the LO-based oleogel has a higher G′ value, indicating that it possesses greater strain stability. This suggests that the gel network formed by intermolecular interactions in the LO-based oleogel is stronger, leading to the extension of the linear viscoelastic region. The point at which the transition occurs from the elastic deformation region to the plastic deformation region, as the strain value increases, is known as the linear yield stress (γ0). For the LO-based oleogel, γ0 is 0.04, while for the CO-based oleogel, it is 0.02 (Table S3). This indicates that the LO-based oleogel is less susceptible to changes in stress values, suggesting a stronger gel strength than other oleogels. As the stress values exceed the LVR range, both the average G′ and G″ values decrease, indicating a gradual breakdown of the gel’s network structure. Once the stress value surpasses the critical stress value (λc), where G′ = G″, all samples exhibit elastic softening and viscosity thinning, resulting in a plastic flow. This is consistent with the results of Figure S4. The λc values for the LO-based and CO-based oleogels are 19.95 and 1.26, respectively (Table S3). This suggests that oleogels formed by oils rich in saturated fatty acids are more prone to fracture under lower strain conditions [48]. Interestingly, for the CO-based oleogel, the G′ value suddenly decreases under high stress. This suggests that the oleogel exhibits excellent spreadability, making it an ideal choice for novel edible coatings [49]. In addition, comparing the stress–strain curves of oleogels prepared from different types of oils reveals that the strain value increases with an increase in the degree of unsaturation.

Frequency sweeps (Figure 5c) at a preparation temperature of 120 °C reveal that, except for the CO-based oleogel, all other oleogels exhibit G′ > G″ behavior. At high-frequency values, there are no crossover points, indicating strong oleogel characteristics. Calculation using the power-law model (Equation (6)) yields relatively large “a” values and small “b” values (Table S4), reflecting good deformation stability of the oleogels. The G′ values of the oleogels remain unaffected by frequency. However, the CO-based oleogel is the only one showing frequency dependence and a high “b” value, indicating the weakest oleogel structure. Moreover, computing the loss tangent (tanδ = G″/G′) can provide additional evidence for the overall viscoelastic behavior of the oleogels (Figure 5d). At a frequency of 0.1 Hz, the tanδ values for the LO-based and CO-based oleogels are 0.099 and 1.23, respectively. This indicates that a higher degree of unsaturation and a greater content of polyunsaturated fatty acids (PUFAs) can enhance the strength of the crystal network within the oleogel [50]. These findings further support the idea that the internal network of the oleogels consists of non-covalent interactions, forming a physically crosslinked gel network. Additionally, the CO-based oleogel exhibits the lowest G′ value, which can be attributed to the formation of a rod-like crystal structure through the combination of saturated fatty acids and β-amyrin. This observation aligns with the micrographs of the oleogel and the relative crystallinity values obtained from XRD analysis (Table S2).

### 3.6. Correlation Analysis of Oleogels

**Figure 6:**
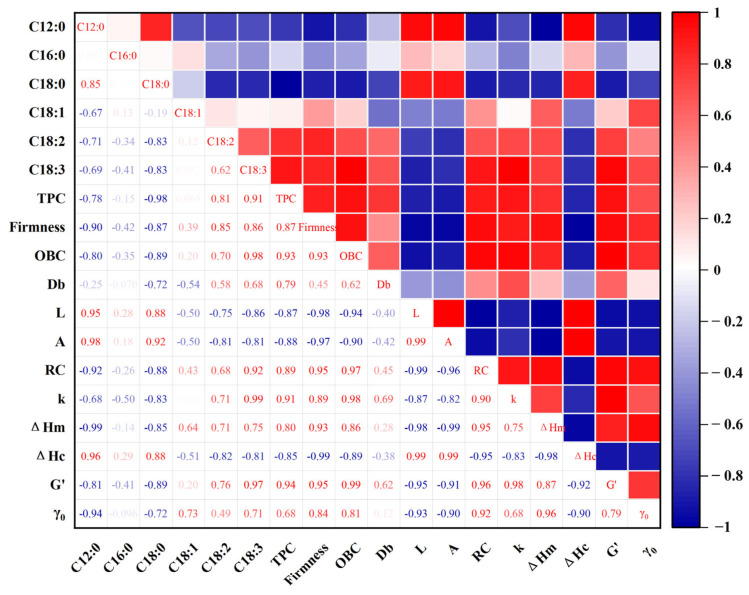
Correlation plots of key parameters (blue indicates negative correlation, red indicates positive correlation).

Through correlation analysis, a deeper understanding of the influence of different oil types on the characteristics of oleogels can be gained. From the perspective of fatty acids, C18:3 shows a positive correlation with OBC (R = 0.97), RC (R = 0.83), and G′ (R = 0.92). Additionally, the increase in hardness, OBC, RC, and G′ value is positively correlated with the degree of lipid unsaturation (Figure 6). On the other hand, C12:0 exhibits a negative correlation with ΔHm (R = −0.98) and hardness (R = 0.71). This result indicates that oleogels prepared from longer-chain and highly unsaturated oils exhibit higher strength, whereas oleogels prepared from oils with shorter chains and higher saturation levels have lower strength. The potential reason is that an LO-based oleogel rich in long-chain and unsaturated fatty acids can facilitate interactions between β-amyrin particles [45]. Simultaneously, the result implies that oleogels with higher rheological properties exhibit strong gel strength, characterized by high stability and a solid texture [51]. On the other hand, there is a strong negative correlation between L and RC (R = −0.94), OBC (R = −0.81), and hardness (R = −0.89). Conversely, L shows a positive correlation with A (R = 0.94). These findings suggest that crystal size plays a role in influencing the ability of β-amyrin to bind liquid oil, thereby impacting the texture of the oleogel. An increase in the void fraction is associated with a larger crystal size in the oleogel. The negative correlation between crystal size and gel strength in oleogels has been substantiated in various research studies [33].

## 4. Conclusions

This study represents the first utilization of β-amyrin as a gelator to construct plant oil-based oleogels, demonstrating the feasibility of using β-amyrin alone as a gelator. Additionally, we investigated the physicochemical properties of β-amyrin-based oleogels prepared at different temperatures and with different oil types, aiming to evaluate the influence of oil type and preparation temperature on gel characteristics. The results revealed that β-amyrin has the capability to impart specific structure to oils and form oleogels, with a critical concentration as low as 2–4%. The gel properties were found to be influenced by the preparation temperature as well as the chain length and degree of unsaturation of the lipids. Especially at higher suitable preparation temperatures, lipids with longer-chain unsaturated fatty acids can form stable oleogels when combined with β-amyrin. Through polarized light microscopy, storage stability, physical properties, and rheological results, it is evident that LO-based oleogels exhibit small and dense β-amyrin crystals, resulting in the highest gel strength and stronger storage stability. In contrast, in CO-based oleogels, crystal aggregation leads to weaker gel strength and poorer stability. Correlation analysis indicates a positive correlation between C18:3 and hardness, OBC, and G′, while C12:0 shows a negative correlation with G′. These findings hold promising practical applications as they allow for the design of stable organic oleogels with varying mechanical properties by modifying the preparation conditions and lipid types. This provides the latest insights for the utilization of pentacyclic triterpenes as bioactive compounds in food applications.

## Figures and Tables

**Table 1:** Average length fractal dimension (Db), average length (L), and lacunarity (A) of microstructures in oleogels prepared with different types of oils. Different lowercase letters within the same column indicate significant differences between samples of different oil types at the same preparation temperature (*p* < 0.05). Different uppercase letters within the same column indicate significant differences between samples of the same oil type at different temperatures (*p* < 0.05).

Types	Temperature (°C)	Db	L (μm)	A
LO	80	1.27 ± 0.03 Ca	35.08 ± 1.15 Ad	0.57 ± 0.00 Ab
	100	1.38 ± 0.05 Ba	30.96 ± 1.70 Ad	0.25 ± 0.02 Bd
	120	1.46 ± 0.02 Aa	21.09 ± 6.56 Bc	0.08 ± 0.00 Cd
CO	80	0.82 ± 0.03 Cc	61.10 ± 2.82 Aa	0.86 ± 0.02 Aa
	100	0.97 ± 0.04 Bd	52.10 ± 6.52 Aa	0.85 ± 0.01 Aa
	120	1.07 ± 0.02 Ad	50.54 ± 6.14 Aa	0.78 ± 0.03 Ba
SO	80	1.02 ± 0.01 Ca	42.54 ± 1.21 Ac	0.59 ± 0.04 Ab
	100	1.27 ± 0.05 Ab	35.05 ± 0.72 Bcd	0.36 ± 0.02 Bcd
	120	1.23 ± 0.12 Bbc	31.23 ± 1.28 Bbc	0.13 ± 0.04 Cc
RO	80	1.07 ± 0.04 Bb	45.59 ± 6.96 Abc	0.84 ± 0.02 Aa
	100	1.11 ± 0.02 Ac	41.08 ± 1.55 ABbc	0.45 ± 0.02 Bc
	120	1.29 ± 0.06 Ab	30.35 ± 5.83 Bbc	0.29 ± 0.03 Cbc
PO	80	0.85 ± 0.04 Bc	50.74 ± 2.67 Ab	0.86 ± 0.02 Aa
	100	1.06 ± 0.07 Acd	46.89 ± 2.34 ABab	0.68 ± 0.08 Bb
	120	1.14 ± 0.23 Ac	36.43 ± 8.57 Bb	0.28 ± 0.02 Cb

**Table 2:** Modified Avrami model parameters of oleogels formed by different oil types and β-amyrin as a function of their crystallization cooling rates.

	5 °C min^−1^			10 °C min^−1^		
Type	n	K (min^−1^)	R^2^	n	K (min^−1^)	R^2^
LO	2.96 ± 0.13	0.03 ± 0.02	0.95 ± 0.02	3.03 ± 0.47	0.07 ± 0.03	0.98 ± 0.01
CO	2.75 ± 0.19	ND	0.85 ± 0.02	2.80 ± 0.02	0.02 ± 0.01	0.87 ± 0.00
SO	2.59 ± 0.18	0.01 ± 0.00	0.96 ± 0.01	2.66 ± 0.32	0.03 ± 0.02	0.98 ± 0.01
RO	2.79 ± 0.07	0.02 ± 0.00	0.98 ± 0.02	2.97 ± 0.30	0.02 ± 0.01	0.97 ± 0.02
PO	2.75 ± 0.12	ND	0.97 ± 0.01	2.31 ± 0.07	0.01 ± 0.01	0.90 ± 0.11

## Data Availability

The original contributions presented in the study are included in the article/Supplementary Material, further inquiries can be directed to the corresponding authors.

## Supplementary Materials

The following supporting information can be downloaded at: https://www.mdpi.com/article/10.3390/foods13060876/s1, Figure S1: Relationship between hardness and preparation temperature, Figure S2: XRD pattern of β-amyrin, Figure S3: XRD patterns of oleogels. (A) XRD patterns of different edible oils, (B–D) prepa-ration temperatures of 80 °C, 100 °C and 120 °C respectively, Figure S4: Viscosity profiles of β-amyrin-based oleogels prepared with different oil types; Table S1: FO, CO, SO, RO, PO (g/100 g) Major fatty acid composition and total polar fraction (TPC%), Table S2: Relative crystallinity (%) of oleogels at different preparation temperatures, Table S3: Thermal behavioral characteristics of oleogels prepared with different oil types with 4% β-amyrin, Table S4: Physical properties of the oleogels. Including linear ultimate stress (γ0), supercritical strain values (λc, stress values for G′ = G″), and (G′ = a ωb) under the power-law exponent where a(Pa⋅b) and b are dimensionless flow behavior exponent.
