# Supplementary material for: The Impact of Oil Type on the Performance of β-Amyrin-Based Oleogels: Formation, Physicochemical Properties, and Potential Correlation Analysis
Source: Foods. 2024 Mar 14;13(6):876. doi: 10.3390/foods13060876 (PMC10969853; doi:10.3390/foods13060876)
Supplement: Supplementary file 1 [file foods-13-00876-s001.zip › foods-2917514-supplementary.pdf]

Supplementary Figures

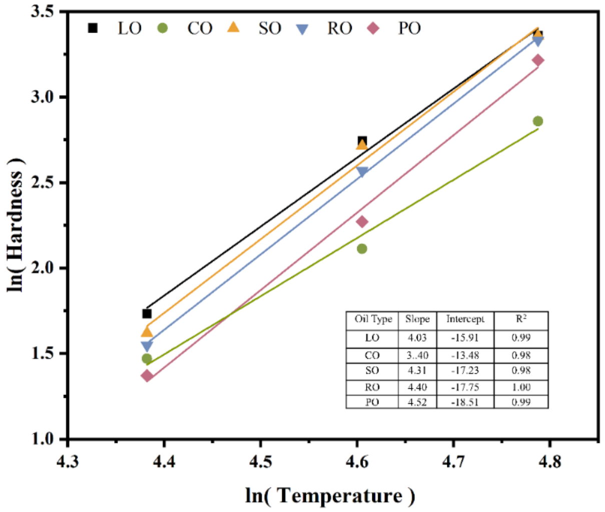

Figure S1: Relationship between hardness and preparation temperature.

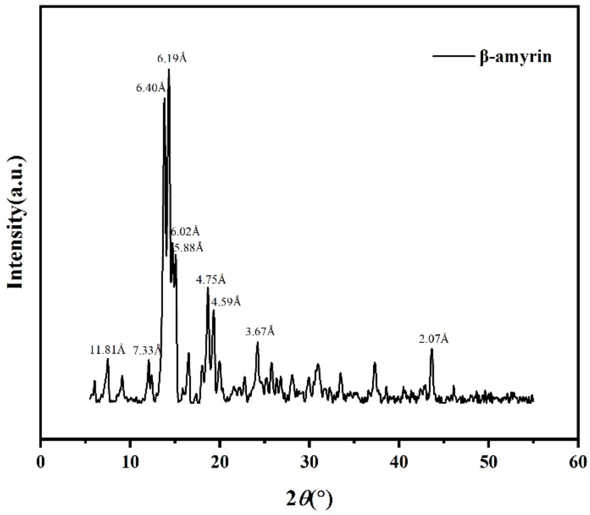

Figure S2:XRD pattern of β-amyrin

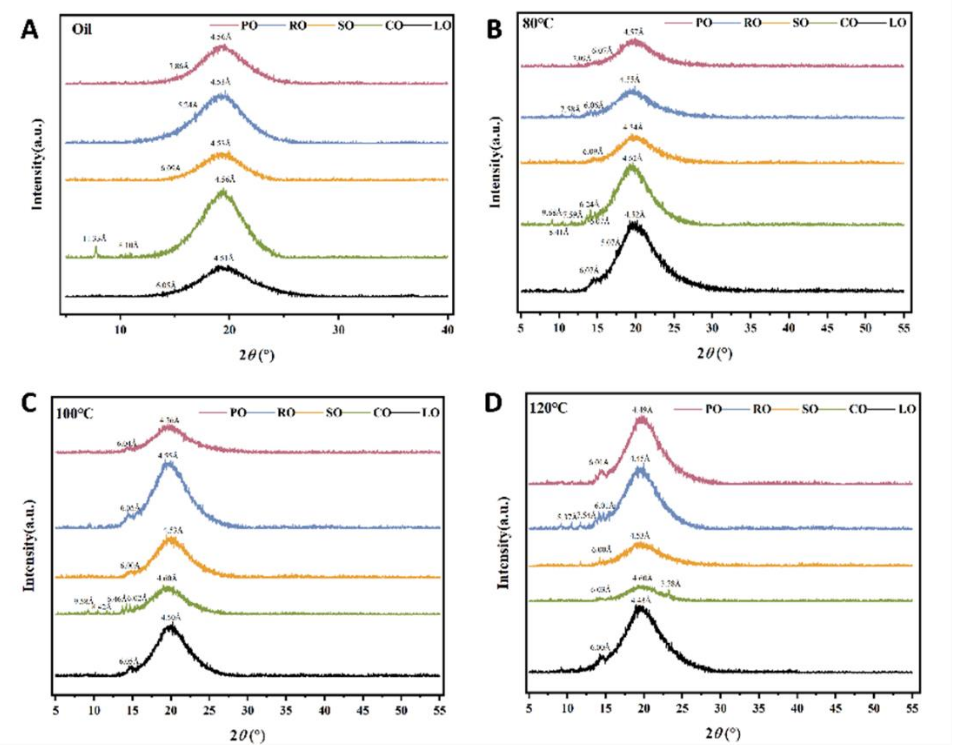

Figure S3: XRD patterns of oleogels. (A) XRD patterns of different edible oils.(B-D) preparation temperatures of 80°C, 100°C and 120°C respectively.

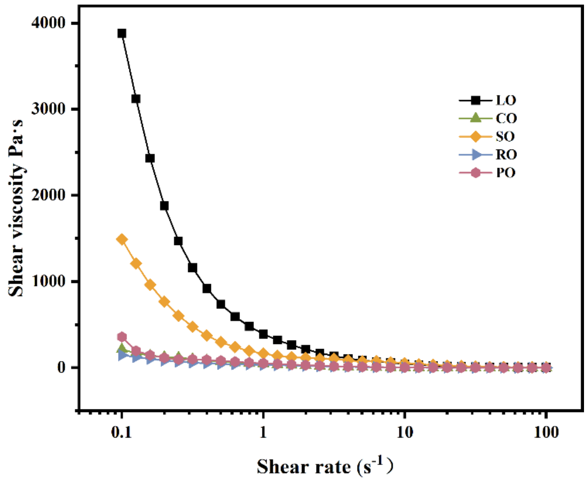

Figure S4: Viscosity profiles of β-amyrin-based oleogels prepared with different oil types

Supplementary Tables

Table S1: FO, CO, SO, RO, PO (g/100 g) Major fatty acid composition and total polar fraction (TPC%)

**Types**	**Fatty acid Structure (%)**							**TPC(%)**
	**C12：0**	**C14：0**	**C16：0**	**C18：0**	**C18：1**	**C18：2**	**C18：3**	
LO	0	0.03	5.06	0	21.03	20.43	52.00	16
CO	49.1	21.8	6.4	1.20	0	6.1	1.2	5
SO	0.03	0.1	6.31	3.54	27.95	61.62	0.29	12
RO	0	0.05	3.63	1.10	76.35	12.20	1.10	4
PO	0.13	0.79	37.05	0.16	45.93	10.43	0.16	11

Table S2: Relative crystallinity (%) of oleogels at different preparation temperatures

Types	80℃	100℃	120℃
LO	74.42±0.26	77.783±2.195	86.907±2.116
CO	70.34±0.60	70.650±3.120	70.867±2.698
SO	68.14±3.09	72.327±0.080	76.607±0.601
RO	65.49±3.06	75.573±0.703	79.207±3.339
PO	65.580±1.763	70.477±1.419	77.850±1.675

Table S3: Thermal behavioral characteristics of oleogels prepared with different oil types with 4% β-amyrin

Types	Temperature rate（℃/min）	Heating			Cooling		
		Ton（℃）	Tm（℃）	ΔHm	Ton（℃）	Tc（℃）	ΔHc
LO	5	-33.58±0.03	-29.54±0.13	49.21±0.06	-62.30±0.12	-38.93±0.01	17.04±0.04
	10	-32.85±0.10	-28.03±0.06	51.37±0.03	-59.63±0.27	-39.09±0.14	103.56±0.21
CO	5	7.72±0.13	24.16±0.06	96.64±0.23	-19.81±0.0	3.90±0.05	94.19±0.06
	10	7.81±0.04	25.32±0.08	101.23±0.20	-7.58±0.01	2.15±0.11	157.46±0.17
SO	5	-47.21±0.22	-27.19±0.08	48.14±0.22	-54.00±0.06	-36.39±0.08	11.42±0.05
	10	-41.81±0.08	-26.84±0.09	60.60±0.29	-45.28±0.10	-39.82±0.10	40.26±0.20
RO	5	-36.88±0.02	-21.41±0.08	55.16±0.21	-57.52±0.22	-27.36±0.06	26.58±0.12
	10	-32.61±0.11	-18.34±0.07	55.80±0.45	-56.37±0.07	-27.96±0.31	21.65±0.26
PO	5	-20.84±0.23	3.83±0.00	64.68±0.10	-46.73±0.04	1.68±0.03	51.78±0.11
	10	-19.68±0.03	5.48±0.06	69.00±0.70	-26.06±0.08	1.18±0.11	76.41±0.02

Table S4: Physical properties of the oleogels. Including linear ultimate stress (γ0), supercritical strain values (c, stress values for G'=G"), and (G'=a ω^b) under the power-law exponent where a(Pa · b) and b are dimensionless flow behavior exponent.

Types	a(Pa · s^b)	b	γ_0（%）	λc
LO	13172.37±478.21a	0.04±0.00b	0.04±0.00a	19.95±0.11a
CO	128.98±8.72e	0.15±0.01a	0.02±0.01b	1.59±0.07d
SO	5400.25± 454.32 b	0.05±0.01b	0.02±0.00b	7.95±3.46c
RO	2619.07±104.45d	0.05±0.02b	0.03±0.02a	19.95±0.97a
PO	3102.37± 388.00c	0.05±0.04b	0.02±0.00b	15.85±0.22b
